# A Sound Lodged in the Uterus

**Published:** 1871-12

**Authors:** 


A SOUND LODGED IN THE UTERUS.—Drs. Petreguin and Foltz report the following: A woman allowed a midwife to introduce a sound into her uterus for the purpose of procuring abortion. The sound disappeared in the genitals and could not be found. Abortion followed. About four months later, the woman observed a small tumor near the umbilicus, which proved to be the head of the sound. The os was dilated by means of a sponge-tent, and in the anterior wall of the uterus the other end of the sound could be felt, which had perforated the uterus near the internal os, and had penetrated upward between the bladder and uterus. The handle of the sound could only be felt in the uterine parenchyma when the woman had been walking about some time. Attempts to remove the sound by way of the vagina failed, and it was finally taken away through an incision made into the abdominal parietes. Recovery followed without further disturbance.—*Schmidt's Jahrbucher.*

